# Stimulus-Driven Population Activity Patterns in Macaque Primary Visual Cortex

**DOI:** 10.1371/journal.pcbi.1005185

**Published:** 2016-12-09

**Authors:** Benjamin R. Cowley, Matthew A. Smith, Adam Kohn, Byron M. Yu

**Affiliations:** 1 Machine Learning Department, Carnegie Mellon University, Pittsburgh, Pennsylvania, United States of America; 2 Center for Neural Basis of Cognition, Carnegie Mellon University, Pittsburgh, Pennsylvania, United States of America; 3 Department of Ophthalmology, University of Pittsburgh, Pittsburgh, Pennsylvania, United States of America; 4 Department of Bioengineering, University of Pittsburgh, Pittsburgh, Pennsylvania, United States of America; 5 Fox Center for Vision Restoration, University of Pittsburgh, Pittsburgh, Pennsylvania, United States of America; 6 Dominick Purpura Department of Neuroscience, Albert Einstein College of Medicine, Bronx, New York, United States of America; 7 Department of Ophthalmology and Vision Sciences, Albert Einstein College of Medicine, Bronx, New York, United States of America; 8 Department of Electrical and Computer Engineering, Carnegie Mellon University, Pittsburgh, Pennsylvania, United States of America; 9 Department of Biomedical Engineering, Carnegie Mellon University, Pittsburgh, Pennsylvania, United States of America; University of Connecticut, UNITED STATES

## Abstract

Dimensionality reduction has been applied in various brain areas to study the activity of populations of neurons. To interpret the outputs of dimensionality reduction, it is important to first understand its outputs for brain areas for which the relationship between the stimulus and neural response is well characterized. Here, we applied principal component analysis (PCA) to trial-averaged neural responses in macaque primary visual cortex (V1) to study two fundamental, population-level questions. First, we characterized how neural complexity relates to stimulus complexity, where complexity is measured using relative comparisons of dimensionality. Second, we assessed the extent to which responses to different stimuli occupy similar dimensions of the population activity space using a novel statistical method. For comparison, we performed the same dimensionality reduction analyses on the activity of a recently-proposed V1 receptive field model and a deep convolutional neural network. Our results show that the dimensionality of the population response changes systematically with alterations in the properties and complexity of the visual stimulus.

## Author Summary

A central goal in systems neuroscience is to understand how large populations of neurons work together to enable us to sense, to reason, and to act. To go beyond single-neuron and pairwise analyses, recent studies have applied dimensionality reduction methods to neural population activity to reveal tantalizing evidence of neural mechanisms underlying a wide range of brain functions. To aid in interpreting the outputs of dimensionality reduction, it is important to vary the inputs to a brain area and ask whether the outputs of dimensionality reduction change in a sensible manner, which has not yet been shown. In this study, we recorded the activity of tens of neurons in the primary visual cortex (V1) of macaque monkeys while presenting different visual stimuli. We found that the dimensionality of the population activity grows with stimulus complexity, and that the population responses to different stimuli occupy similar dimensions of the population firing rate space, in accordance with the visual stimuli themselves. For comparison, we applied the same analysis methods to the activity of a recently-proposed V1 receptive field model and a deep convolutional neural network. Overall, we found dimensionality reduction to yield interpretable results, providing encouragement for the use of dimensionality reduction in other brain areas.

## Introduction

Dimensionality reduction has been applied to neural population activity to study decision making [1, 2], motor control [3–5], olfaction [6], working memory [7, 8], visual attention [9], audition [10], rule learning [11], speech [12], and more (for a review, see [13]). In many cases, dimensionality reduction is applied in brain areas for which the relationship between neural activity and external variables, such as the sensory stimulus or behavior, is not well characterized. This is indeed the setting in which dimensionality reduction may be most beneficial because it allows one to relate the activity of a neuron to the activity of other recorded neurons, without needing to assume a moment-by-moment relationship with external variables. However, it is also the setting in which the outputs of dimensionality reduction can be the most difficult to interpret.

To aid in interpreting the outputs of dimensionality reduction in such settings, it is important to vary the inputs to a brain area and ask whether the outputs of dimensionality reduction change in a sensible way. This is most readily done for a brain area close to the sensory periphery, such as the primary visual cortex (V1). Here, we apply dimensionality reduction to V1 and ask two fundamental, population-level questions. First, how is neural complexity related to stimulus or task complexity? Previous studies have used dimensionality reduction to analyze population activity in a reduced space (e.g., [1–13]). Implicit in these studies is the appropriate dimensionality of the reduced space, which is a measure of neural complexity. It is currently unknown how neural complexity scales with stimulus or task complexity for a given population of neurons [14]. Second, how does a neural circuit flexibly encode (or “multiplex”) the representation of the vast number of stimuli encountered in the natural world? Recent studies suggest that it may be possible to take advantage of the multi-dimensional properties of the population activity space [2, 7, 15–17]. In particular, the population activity representing different stimuli might occupy similar dimensions of the population activity space [10]. It is currently unknown how the similarity of the dimensions being occupied by the population activity changes with the similarity of the stimuli.

**Fig 1:**
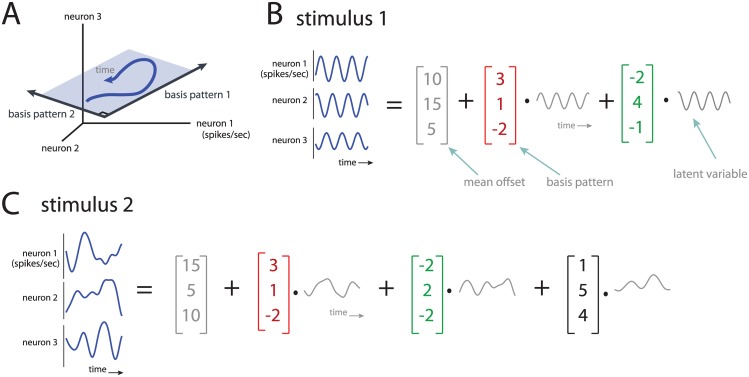
Conceptual illustration of dimensionality and basis patterns. *A*: The activity of three neurons can be plotted in a 3-d population firing rate space, where each axis represents the firing rate of one neuron. The population activity evolves over time (blue trace), and occupies a 2-d plane (blue shade). The basis patterns are orthogonal axes that define this 2-d plane. *B*: The activity of three neurons can also be represented as time-varying firing rates or peri-stimulus time histograms, PSTHs. The activity can be decomposed into a weighted sum of basis patterns (red and green) and a mean offset (gray). Each basis pattern is weighted by a time-varying latent variable. Note that basis patterns are mutually orthogonal, by definition. *C*: The activity of the same three neurons as in *B*, but for a different stimulus. Same conventions as in *B*.

The concept of dimensionality is illustrated in Fig 1. Consider a high-dimensional space (termed the *population firing rate space*) in which each axis represents the firing rate of a recorded neuron (Fig 1*A*). The goal of dimensionality reduction is to identify i) how many dimensions are occupied by the neural population activity, i.e., the *dimensionality* of the population activity, and ii) how these dimensions are oriented within the population firing rate space. In this three-neuron example, the population activity is two-dimensional, where the dimensions are defined by the orthogonal basis patterns (Fig 1*A*, basis patterns 1 and 2). Equivalently, we can think of dimensionality reduction in terms of decomposing the population activity into a weighted sum of basis patterns and a mean offset (Fig 1*B*). A basis pattern describes a characteristic way in which activity of the neurons covaries. Each basis pattern is fixed and is weighted by a time-varying latent variable, which represents the contribution of the basis pattern at each point in time.

We can compare the outputs of dimensionality reduction for two different stimuli (Fig 1*B* and 1*C*). The population activity for stimulus 1 is two-dimensional because it can be described by two basis patterns (Fig 1*B*), whereas that for stimulus 2 is three-dimensional (Fig 1*C*). Thus, the population response to stimulus 2 would be deemed more complex than the population response to stimulus 1. In addition, we can ask whether the population responses to different stimuli occupy similar dimensions within the population firing rate space. This is assessed by comparing the basis patterns across stimuli. In this example, there is one basis pattern that is shared by both stimuli (red), one basis pattern that is similar between stimuli (green), and one basis pattern (black) that is employed only by stimulus 2.

In this work, we characterize how neural complexity varies with stimulus complexity in macaque V1 by applying principal components analysis (PCA) to the trial-averaged neural responses to different classes of visual stimuli, including sinusoidal gratings, a natural movie, and white noise. In addition, we develop a new method (termed the pattern aggregation method) to measure how the basis patterns extracted from the population responses to each stimulus relate to each other. This method allows one to characterize how the similarity of the dimensions being occupied by the population activity changes with the similarity of the stimuli. A key advantage of studying these questions in V1 is that there are well-established receptive field (RF) models. By applying the same dimensionality reduction methods to the outputs of an RF model, we can more deeply understand the relationship between the outputs of dimensionality reduction and known properties of V1 neurons. The results described in this work show that the outputs of dimensionality reduction, when applied to V1 population activity, are sensible, and thus may be fruitfully applied to other brain areas.

## Results

### Dimensionality of population responses to gratings

**Fig 2:**
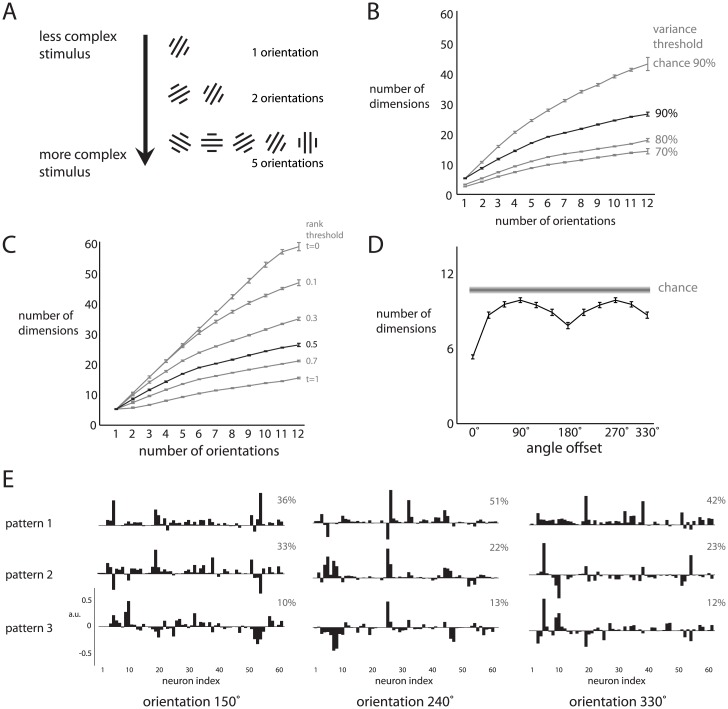
Dimensionality of population responses to individual gratings. *A*: The complexity of the stimulus was varied by combining a different number of consecutive orientations. The least complex stimulus consisted of a single orientation, and more complex stimuli included two or five orientations. *B*: Dimensionality of population activity versus number of orientations. Bottom three curves correspond to the number of dimensions needed to explain 70%, 80%, and 90% of the variance. Top gray curve corresponds to the number of dimensions expected by chance for the 90% variance threshold. Error bars represent the standard error across monkeys and all possible combinations of consecutive orientations. *C*: Varying the rank threshold for a fixed variance threshold (90%). Same data as in *B*. Each curve represents the dimensionality of the population response as the number of consecutive orientations varies, for a particular rank threshold *t*. Error bars are computed as in *B*. *D*: Dimensionality of population activity versus angle offset between two orientations (bottom black curve). Chance dimensionality (top gray curve) and error bars are computed as in *B*. *E*: Basis patterns describing the largest percentage of variance for the population responses to three example orientations (90° apart) for one monkey. Each pattern is a unit vector with a norm of 1. Percentages denote the percent variance explained by each pattern.

We first investigated how changing the complexity of the visual stimulus changes the dimensionality of trial-averaged population responses using drifting sinusoidal gratings. To change the stimulus complexity, we included different numbers of consecutive grating orientations in the analysis (ranging from 1 to 12 orientations). For example, the least complex stimulus included a single orientation, and more complex stimuli included two or five consecutive orientations (Fig 2*A*).

We asked how quickly the dimensionality of the population activity grows as the number of orientations increases. At one extreme, it may be that the population response to each orientation uses an entirely different set of basis patterns (i.e., dimensions). In this case, the dimensionality for two orientations would be two times the dimensionality for one orientation. At the other extreme, it may be that the population response to each orientation resides in the same set of dimensions. In other words, the population response is formed using the same basis patterns, but linearly combined using different weights for different orientations. In this case, the dimensionality for two orientations would be the same as the dimensionality for one orientation.

We first computed the basis patterns of each orientation individually by applying PCA to the trial-averaged population response (taken in 20 ms bins), and identifying the patterns explaining the greatest variance in the population response (up to a chosen cumulative variance threshold, e.g., 90%). To assess the dimensionality of the population response to multiple orientations, we developed the pattern aggregation method (see Methods), which first aggregates the basis patterns for different orientations as column vectors in a matrix, and then computes the number of linearly independent columns of the aggregated matrix (i.e., the effective rank). This value is the dimensionality for multiple orientations. Using a 90% variance threshold, we found that the dimensionality for two orientations was 1.62 times the dimensionality for one orientation (Fig 2*B*, ‘90%’ curve), and significantly smaller than what would be expected had the basis patterns been randomly chosen (Fig 2*B*, ‘chance 90%’ curve, *p* < 10^−5^). In other words, for consecutive orientations separated by 30°, the population responses share about half of their basis patterns. As more orientations were included, the dimensionality of population responses remained lower than expected by chance (Fig 2*B*), indicating that population responses to oriented gratings separated by angles larger than 30° also tend to use similar basis patterns. Similar trends were found using different variance thresholds (Fig 2*B*, ‘70%’, ‘80%’ curves), so we use a 90% threshold in the rest of this work.

The pattern aggregation method requires a choice for the rank threshold *t* to determine how different basis patterns need to be before they define separate dimensions. We repeated the above analysis for different choices of *t* and a fixed variance threshold of 90% (Fig 2*C*). We found that the dimensionality trends are similar for rank thresholds *t* near 0.5, so we use *t* = 0.5 in the rest of this work. Because the absolute dimensionality depends on the variance and rank thresholds, we make no claims about absolute dimensionality. Rather, we focus on relative comparisons of dimensionality for fixed variance and rank thresholds.

We observed a change in the rate of increase of dimensionality after six orientations (Fig 2*B*, black curve). Because consecutive orientations were separated by 30°, the first and seventh orientations were 180° apart and differed only in their drift direction. Thus, the seventh to twelfth orientations were identical to the first to sixth orientations respectively, but drifted in opposite directions. A small proportion of V1 neurons are direction selective [18, 19], so the change in slope of the dimensionality curve might be due to the population activity using similar basis patterns for opposite drift directions.

To test this possibility, we performed two analyses. First, we assessed the direction selectivity of each neuron (direction index = 1 − null response / preferred response), and found that 16 of 183 neurons had a direction index greater than 0.5, consistent with [19]. If none of the neurons encoded direction selectivity, we would expect the dimensionality curve to be flat beyond 6 orientations in Fig 2*B*. The increase in dimensionality after 6 orientations is consistent with the finding that at least some neurons show direction selectivity. A potential concern is that the increase in dimensionality beyond 6 orientations arises from the fact that a larger number of patterns are being aggregated for a larger number of orientations. To address this, we performed a control analysis which equalized the number of patterns being aggregated across different numbers of conditions by including patterns from many subsamples of the data. We found that the dimensionalities for 7 or more orientations were significantly greater than the dimensionality for 6 orientations (*p* < 0.001), following the same trend shown in Fig 2*B*.

Second, we assessed how the dimensionality of the population activity for two orientations varies with the angular offset between the orientations (Fig 2*D*). This indicates how the similarity of the dimensions being occupied by the population activity changes with the similarity of the stimuli. We found that the dimensionality increases with angular offset up to 90°, indicating that the population activity differs the most for two orientations with 90° offset. Then, the dimensionality decreases as the angular offset increases, reaching a minimum at a 180° offset, where gratings drift in opposite directions. Thus, the population activity uses more similar basis patterns for gratings drifting in opposite directions (180° offset) than to gratings of different orientations (angular offsets other than 180°). The dimensionality for 180° offset was higher than that for 0° offset (*p* < 10^−5^), indicating that the population activity does not use identical basis patterns for opposite drift directions. Because these dimensionalities were computed differently (the pattern aggregation method for 180° offset and a variance threshold for 0° offset), we aggregated an equal number of patterns (identified over many subsampled runs) for 0° offset as that for 180° offset, and still found a higher dimensionality for 180° offset than for 0° offset (*p* < 10^−5^).

This result, combined with the change in slope of the dimensionality curve (Fig 2*B*), indicates that the population activity tends to use similar (but not identical) basis patterns for opposite drift directions. Taken together, the analyses in Fig 2*B* and 2*D* characterize how the outputs of dimensionality reduction vary with the sensory input (in this case, drifting sinusoidal gratings) to V1.

We also visualized the basis patterns describing the largest percentage of variance for three different orientations (Fig 2*E*). These basis patterns extracted from the trial-averaged population activity are akin to the hypothetical basis patterns shown in Fig 1 (red, green, black). For a given basis pattern, the absolute height of each bar indicates the degree to which each neuron contributes to that basis pattern. The following are two salient properties of the identified basis patterns. First, most of the neurons in the recorded population contribute to each basis pattern to some extent. Thus, the basis patterns capture changes in firing rates that are shared broadly across the population, rather than reflecting the activity of only a small number of neurons. Second, the basis patterns capture both positive and negative signal correlations between neurons, where the signal is the phase of the grating at different time points. A basis pattern describes positive signal correlation between a pair of neurons if the neurons have coefficients of the same sign. Conversely, a basis pattern describes negative signal correlation for coefficients of opposite sign. We can relate these basis patterns to the results shown in Fig 2*B*–2*D* by asking how similar are the linear combinations of each set of basis patterns across different stimulus orientations. This is difficult to assess by eye, and so we rely on the quantifications shown in Fig 2*B*–2*D* to determine how similar are the basis patterns across stimulus orientations.

### Dimensionality of population responses to different classes of visual stimuli

**Fig 3:**
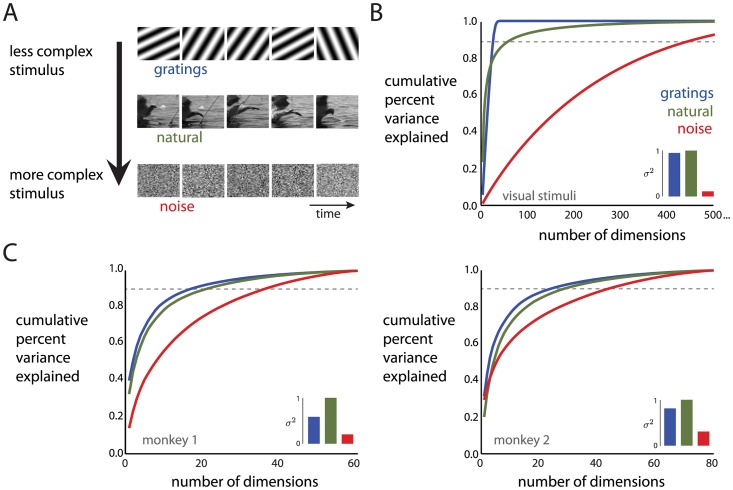
Dimensionality of movie stimuli and population responses to those movies. *A*: Example frames of the movies for the gratings movie, natural movie, and noise movie. *B*: For each stimulus, cumulative percent variance of the stimulus pixel intensities explained by different numbers of dimensions. Dashed line corresponds to 90% of the variance explained. Inset: summed variance of the pixel intensities, normalized by the maximum variance across movies. *C*: For each stimulus, cumulative percent variance of the population activity explained by different numbers of dimensions. Dashed lines correspond to 90% of variance explained. Left panel: monkey 1, 61 neurons. Right panel: monkey 2, 81 neurons. Insets: summed variance of each neuron’s activity, normalized by the maximum variance across movies.

We next sought to determine how the dimensionality of the trial-averaged population activity varies with the complexity of the visual stimulus for a wider range of stimuli. We presented three movie stimuli (Fig 3*A*): a sequence of sinusoidal gratings (‘gratings movie’), contiguous natural scenes (‘natural movie’), and white noise (‘noise movie’). In contrast to Fig 2*A* where the order of stimulus complexity is clear (i.e., a larger number of orientations is more complex), here we needed first to assess the relative complexity of the three movie stimuli. By applying PCA to the pixel intensities, we found that 40 dimensions could explain nearly 100% of the variance of the pixel intensities for the gratings movie (Fig 3*B*). For the natural movie, the top few dimensions explained a large percentage of the variance due to global luminance changes caused by zooming and panning the camera, and a large number of additional dimensions were needed to explain the remaining variance. For the noise movie, each dimension explained only a small percentage of the total variance. We summarized these cumulative percent variance curves by finding the number of dimensions (gratings movie: 24, natural movie: 64, noise movie: 459) needed to explain 90% of the variance (Fig 3*B*, dashed line). Based on these values, the gratings movie was least complex, followed by the natural movie, then the noise movie. Similar results were obtained when first transforming the pixel intensities using a V1 receptive field model, then applying PCA (see “Comparing to a V1 receptive field model”). We further asked how much the pixel intensities varied for each movie stimulus, and found that the noise movie had a smaller variance than the other two movie stimuli (Fig 3*B*, inset). Together, this indicates that the distribution of pixel intensities for the gratings movie and natural movie is akin to an elongated ellipsoid (low dimensionality, high variance), whereas that for the noise movie is akin to a small ball (high dimensionality, low variance).

Having measured the relative complexity of the movie stimuli, we then asked how the dimensionality of the population responses to the movie stimuli varies with stimulus complexity. We found that for a 90% variance threshold, the dimensionality of the trial-averaged population responses (20 ms bins) was ordered in the same way as the stimulus complexity (Fig 3*C*); namely, the population responses to the gratings movie had the lowest dimensionality, followed by the natural movie, then the noise movie. This ordering did not simply follow from the ordering of the mean population firing rates for the different movies (monkey 1: 4.2, 6.4, 5.4 spikes/sec, monkey 2: 6.6, 8.2, 6.7 spikes/sec for gratings, natural, and noise movies, respectively), and was consistent for a wide range of neuron counts for both monkeys (S1 Fig). We also assessed how much the firing rates varied in response to each movie stimulus—that is, we measured how much the mean firing rate (averaged across experimental trials) varies over time. As with pixel intensities, we found that the population response to the noise movie had the smallest variance, followed by the gratings movie, then the natural movie (Fig 3*C*, inset). Overall, the dimensionality and variance ordering in the visual stimuli and the population responses were similar, indicating that the population activity in V1 retains the complexity of the ordering of the visual stimuli themselves.

### Basis patterns of population responses

Having compared the dimensionality of the population activity across stimuli, we next asked how the basis patterns of the population activity (corresponding to the dimensions being occupied by the population activity) compare across stimuli. Previous studies have found that the ability of a RF model to predict a neuron’s response can depend on the stimulus class on which the model was trained [20–22], suggesting that the population activity might use somewhat distinct basis patterns for different stimulus classes. On the other hand, if basis patterns are influenced by the shared underlying network structure [4, 10], then we would expect them to be shared across responses to different stimuli.

**Fig 4:**
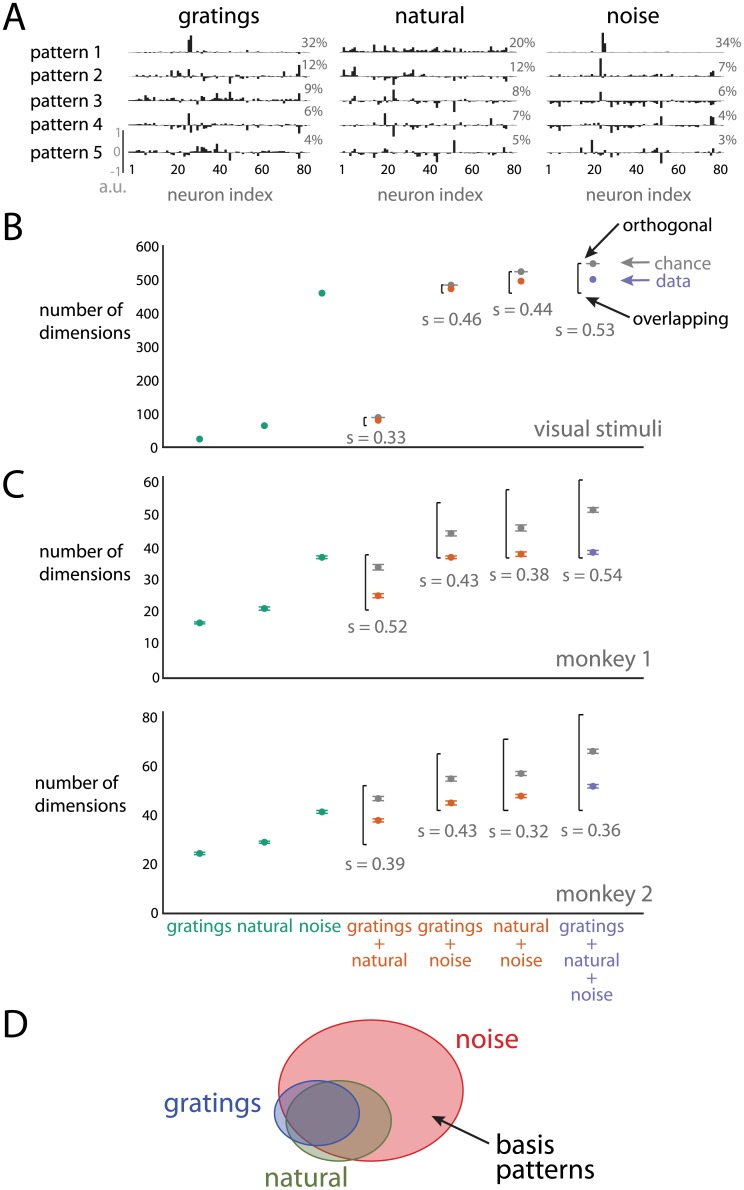
Similarity of basis patterns across stimuli. *A*: Basis patterns describing the largest percentage of variance for the population responses to the gratings, natural, and noise movies for monkey 2. Each pattern is a unit vector with a norm of 1. Percentages denote percent variance explained by each pattern. *B*: Dimensionality of visual stimuli for individual movies (teal dots), and combinations of two (orange dots) or three (purple dots) movies. The teal dots correspond to where the curves intersect the dashed lines in Fig 3*B*. Black brackets denote the range of possible dimensionalities (bottom of the black bracket corresponds to overlapping patterns; top of the black bracket corresponds to orthogonal patterns). Gray dots indicate dimensionalities expected by chance, and error bars represent the standard deviation of 100 random samples. The similarity index *s* indicates if patterns overlap more than expected by chance (*s* > 0) or are closer to orthogonal than expected by chance (*s* < 0). *C*: Dimensionality of population activity, for individual and combinations of movies. Same conventions as in *B*. Error bars represent standard deviations of the subsampled estimates. *D*: Venn diagram that summarizes the similarity of basis patterns across stimuli. The size of each ellipse indicates the number of basis patterns, and the overlap indicates the extent to which basis patterns are shared by different stimuli.

We first asked whether there are qualitative differences in the coefficients of the basis patterns for population responses to the stimulus movies (Fig 4*A*). As in Fig 2*E*, we found that most of the basis patterns represented activity across a large number of neurons and described both positive and negative signal correlations. However, there were two notable exceptions. First, the basis pattern describing the most variance for the population response to the gratings and noise movies involved primarily two neurons (Fig 4*A*, right and left panels, pattern 1, neuron indices 26 and 27). For these movies, the two neurons had the highest firing rate modulation (maximum—minimum firing rate) across the recorded population. The weights for the gratings movie appeared to be sparser than those for individual gratings (Fig 2*E*), likely because the gratings movie contained different orientations that strongly co-modulated these similarly-tuned neurons, whereas individual gratings with a single orientation co-modulated many neurons together with phase. Second, the basis pattern describing the most variance for the population response to the natural movie had coefficients of the same sign (Fig 4*A*, middle panel, pattern 1). This is due to the entire population increasing or decreasing its firing rates together in response to global luminance changes prevalent in natural movies. Other than the similarity of the top basis pattern for the gratings and noise movies, it was difficult to determine by eye whether the basis patterns were being shared across stimuli. Thus, we used the pattern aggregation method to quantitatively assess the similarity of the identified basis patterns.

### Assessing similarity of basis patterns

As a baseline, we assessed the extent to which the visual stimuli themselves reside in the same dimensions in pixel space. We compared the dimensionalities of the individual movies (Fig 4*B*, teal dots, consistent with Fig 3*B*) to those of combinations of movies (Fig 4*B*, orange and purple dots). If the stimuli reside in overlapping dimensions, then the resulting dimensionality would be the maximum of the dimensionalities for the individual movies. However, if the stimuli reside in completely non-overlapping (i.e., orthogonal) dimensions, then the resulting dimensionality would be the sum of the dimensionalities for the individual movies. To measure the extent of overlap, we computed a similarity index *s*, for which *s* > 0 indicates that the patterns are more similar (i.e., more overlapping) than expected by chance and *s* < 0 indicates that the patterns are less similar (i.e., closer to orthogonal) than expected by chance (see Methods). There are two main observations. First, the dimensions occupied by the gratings movie overlap with those for the natural movie. To see this, note that the dimensionality for the gratings and natural movies together (80 dimensions) was less than the sum of the individual dimensionalities for the two movies (Fig 4*B*, gray, 88 dimensions). To ensure that the overlap in patterns was meaningful, we confirmed that the aggregated dimensionality of 80 was less than the dimensionality (88 dimensions) that would be expected by combining randomly-chosen dimensions (*s* = 0.33, *p* < 10^−5^). Second, the dimensions occupied by the noise movie include many of the dimensions for the other two stimuli. This is indicated by the fact that the dimensionality corresponding to any combination of stimuli that included the noise movie (gratings + noise: 472, natural + noise: 495, and gratings + natural + noise: 500 dimensions) was less than the dimensionality that would be expected by chance (*s* > 0.4, *p* < 10^−5^ for all cases). Note that, in all cases, the chance dimensionality was near the maximum dimensionality (indicating orthogonality between the two sets of randomly-chosen patterns) because the dimensionality of the pixel space (1,600 dimensions) was much larger than the dimensionalities of the individual movies.

We used the same approach to analyze the population responses as we did the visual stimuli. We compared the dimensionality of the population responses to individual movies (Fig 4*C*, teal dots, consistent with Fig 3*C*) to that of population responses to combinations of movies (Fig 4*C*, orange and purple dots). We found that the relationship of the basis patterns employed by the population activity across stimuli (Fig 4*C*) was similar to the relationship between the stimuli themselves (Fig 4*B*). First, the dimensions occupied by the population responses to the gratings movie are overlapping with those for the natural movie. This is indicated by the fact that the aggregated dimensionality for the population responses to the gratings and natural movies (monkey 1: 25, monkey 2: 38) was less than the dimensionality if the patterns were orthogonal (top of the black brackets) and the dimensionality expected by chance (*s* > 0.39, *p* < 10^−5^). Second, the dimensions occupied by the population response to the noise movie include most of the dimensions for the other two stimuli. This is indicated by the fact that the dimensionality corresponding to any combination of stimuli that included the noise movie was less than the dimensionality expected by chance (*s* > 0.32, *p* < 10^−5^ in all cases), and close to entirely overlapping.

The chance dimensionality in Fig 4*C* is computed by assuming that any population activity pattern within the *N*-dimensional population firing rate space can be achieved (where *N* = 61 for monkey 1 and *N* = 81 for monkey 2). Previous studies indicate that the population activity may only be able to occupy a subset of dimensions due to underlying network constraints [4, 10]. Although we had no way of identifying exactly how many dimensions could have been occupied by the population of recorded neurons, we computed a lower bound by determining *M*, the largest value of dimensionality observed in response to any combination of stimuli (Fig 4*C*, *M* = 39 for monkey 1 and *M* = 52 for monkey 2). If the chance level is computed instead by drawing random patterns from this *M*-dimensional space, we still find that the population activity tends to occupy more similar dimensions than expected by chance for all pairs of movies (*p* < 0.05). This is a conservative assessment because larger values of *M* would only make the comparison more statistically significant. We note that even if the population responses to different stimuli occupy similar dimensions, this does not imply that the responses occupy the same regions of the subspace defined by those dimensions. In other words, the activity may covary along the same dimensions but be centered at different locations in the population activity space. We found that the population responses to the three movies indeed were centered in different locations of the population activity space (S2 Fig).

Our analysis of the similarity of basis patterns for the population activity is summarized by the schematic in Fig 4*D*, where the size of each ellipse represents the dimensionality (i.e., number of basis patterns) of the population response to the corresponding stimulus and the overlap between ellipses represents the extent to which the population responses share basis patterns. We found that the basis patterns for the gratings movie were overlapping with those of the natural movie, and that the basis patterns for the noise movie largely contain the basis patterns for the other two stimuli. However, there were a small number of basis patterns that were unique to each stimulus, shown by the small areas of non-overlap among the ellipses. Overall, this suggests that a neural circuit is capable of expressing a limited repertoire of basis patterns, and that a subset of those basis patterns is employed for any given stimulus.

### Time-resolved dimensionality of population responses to movie stimuli

In the preceding sections, the analyses of the movie stimuli and the corresponding neural activity used the entire 30-second movie (i.e., 750 time points) together. Here, we consider time-resolved measurements of dimensionality using one second windows, each comprising 25 time points. This allows us to assess how dimensionality changes over time, and compare the basis patterns employed by the trial-averaged population activity during different parts of the 30-second movies.

**Fig 5:**
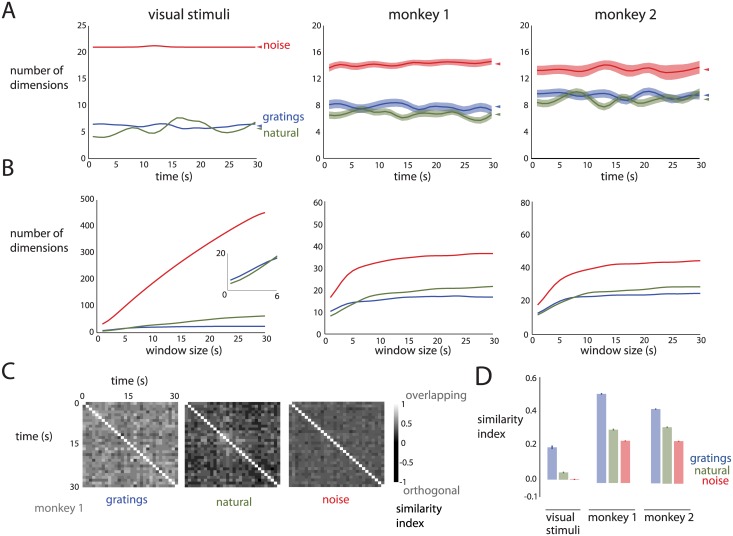
Time-resolved dimensionality of movie stimuli and population responses. *A*: Dimensionality versus time. Left panel: visual stimuli. Center panel: monkey 1 (61 neurons). Right panel: monkey 2 (81 neurons). Error bars are standard deviations of subsampled estimates. Triangles denote mean dimensionality across time for each movie. Curves were smoothed with a Gaussian kernel with a standard deviation of 1.5 s. *B*: Dimensionality with growing time windows starting at the beginning of each movie. Left panel: visual stimuli. Inset: zoomed portion of the bottom-left of the plot. Center panel: monkey 1. Right panel: monkey 2. Curves were smoothed as in *A*. Due to the smoothing, the dimensionalities for the 1 second window do not exactly match the leftmost point in *A*, and those for the 30 second window do not exactly match the dimensionalities in Fig 3. *C*: Similarity of the basis patterns employed by the population responses across time (monkey 1). Each element in the similarity matrix corresponds to the similarity index between the sets of patterns for a pair of time points. *D*: Mean similarity index for visual stimuli and population responses. Error bars denote standard error over similarity indices.

For the visual stimuli (Fig 5*A*, left panel) and the population responses (Fig 5*A*, center and right panels), we found that the dimensionality corresponding to the noise movie was higher than the dimensionality corresponding to the gratings and natural movies in each one-second window, consistent with the results of analyzing each 30-second movie in its entirety (Fig 3). However, the dimensionality of the natural movie was not greater than that of the gratings movie (cf. colored triangles in Fig 5*A*, which indicate average dimensionality over time), in contrast to Fig 3. We hypothesized that this was due to temporal correlations in the natural movie, in which frames within a one-second window tend to be self-similar. In contrast, for the gratings and noise movies, there are at least three different grating orientations and 25 different frames of white noise within each one-second window.

To reconcile the results for short and long time windows, we performed three analyses. First, we tested the hypothesis that temporal correlations in the natural movie result in lower dimensionalities (relative to the other movies) for short time windows. To break the temporal correlations, we shuffled the time points (20 ms resolution) across each 30 second period and performed the same analysis as in Fig 5*A*. We found that, in a one-second window, the mean dimensionality corresponding to the natural movie was higher than that corresponding to the gratings movie for the shuffled data. This was true for the visual stimuli (*p* < 0.01) and for the population responses (monkey 1: *p* < 10^−3^, monkey 2: *p* < 0.05). This indicates that the range of basis patterns expressed by the population responses to the natural movie within a short time window is limited by the temporal correlations in the natural movie itself.

Second, we asked how the dimensionality grows when increasing the window size progressively from one second to 30 seconds, where each window starts at the beginning of the movie (Fig 5*B*). If the dimensionality increases with window size, this would indicate that new patterns (of pixels or of population activity) are being used throughout the duration of the movie. However, if the dimensionality plateaus, then the patterns are being reused and no new patterns are being expressed. The leftmost points on these curves (one-second window) correspond to the leftmost points on the corresponding curves in Fig 5*A*. The rightmost points on these curves (30-second window) correspond to the dimensionalities shown in Fig 3. Although the dimensionality corresponding to the natural movie (green) is lower than that corresponding to the gratings movie (blue) for short time windows, the dimensionality corresponding to the natural movie grows more quickly and surpasses the dimensionality corresponding to the gratings movie for longer time windows. We found this to be true for both the visual stimuli (Fig 5*B*, left panel) and the population responses (Fig 5*B*, center and right panels). This indicates that the patterns for the natural movie tend to be self-similar within a short time window, and new patterns are expressed over longer time windows. In contrast, the dimensionality corresponding to the gratings movie does not grow as quickly with window size. This is because once a few different grating orientations are included, patterns corresponding to additional grating orientations can be represented approximately as linear combinations of patterns corresponding to existing grating orientations (both for the visual stimuli and the population responses), and therefore do not increase the dimensionality appreciably.

Third, we directly measured the similarity of the patterns between different one-second windows using the pattern aggregation method. Fig 5*C* shows the matrix of similarity indices for the population responses to each of the three movies (monkey 1). By averaging the values of the off-diagonal similarity indices across the matrix, we found that the patterns corresponding to the gratings movie tended to be more similar across time than those for the natural and noise movies (Fig 5*D*). This was true for the visual stimuli (left panel), as well as for the population responses (middle and right panels). This result indicates that new patterns tend to be expressed (for both the visual stimuli and the population responses) as the movies play out over time more for the natural and noise movies than for the gratings movie. This result also supports the finding in Fig 5*B* that the dimensionality corresponding to the natural movie grows more quickly than that corresponding to the gratings movie.

Taken together, these results indicate that the noise movie drives a large number of basis patterns in the population activity in a short time window, relative to the gratings and natural movies. As the movies play out over time, the noise and natural movies tend to drive new basis patterns, whereas the grating movie tends to recruit the same patterns.

We also asked whether the second-by-second fluctuations of the dimensionality of the visual stimulus during the 30-second movie (Fig 5*A*, left panel) were related to that of the population responses (Fig 5*A*, middle and right panels) and found weak correlations (mean across movies *ρ* = 0.20). Although the dimensionality fluctuations were larger for the natural movie than the other two movies (Fig 5*A*, left panel), these fluctuations were less salient in the population activity (Fig 5*A*, middle and right panels). These two observations underscore that, although there are similarities in the statistical properties of the visual stimuli and population responses, there are also differences that remain to be understood.

### Comparing to a V1 receptive field model

One of the key advantages of performing this study in V1 is that much is known about the stimulus-response relationship of individual neurons, as described by the many RF models that have been proposed in the literature [23]. Although the RF models do not capture every aspect of V1 neuronal activity [20–22, 24], we can apply the same dimensionality reduction methods to the activity generated by an RF model to help interpret the outputs of dimensionality reduction. We consider it a strength that, in many cases (described below), the outputs of dimensionality reduction applied to population activity show the same trends as when applied to activity from an RF model. This similarity indicates that single-neuron properties are reflected in population metrics, such as dimensionality. In cases where there are discrepancies between the outputs of dimensionality reduction for population activity and RF models, our results can provide guidance necessary to improve RF models.

Although a complete study of the many V1 RF models available is beyond the scope of this work, here we focus on one recently-proposed model [25]. The model has four components: a Gabor filter, whose output is half-rectified; an untuned suppressive filter, whose output is also half-rectified; a normalization signal; and an exponentiating output nonlinearity. These components are shared with many other models of V1 [23], and thus make it well-suited for studying how dimensionality changes as the input image is transformed by each component. The parameter values for 100 model neurons were drawn from parameter distributions reported in [25], since we did not present the stimuli appropriate for fitting the model parameters to data. For this reason, we only compare trends between the dimensionality of the outputs from the model and that of population activity.

**Fig 6:**
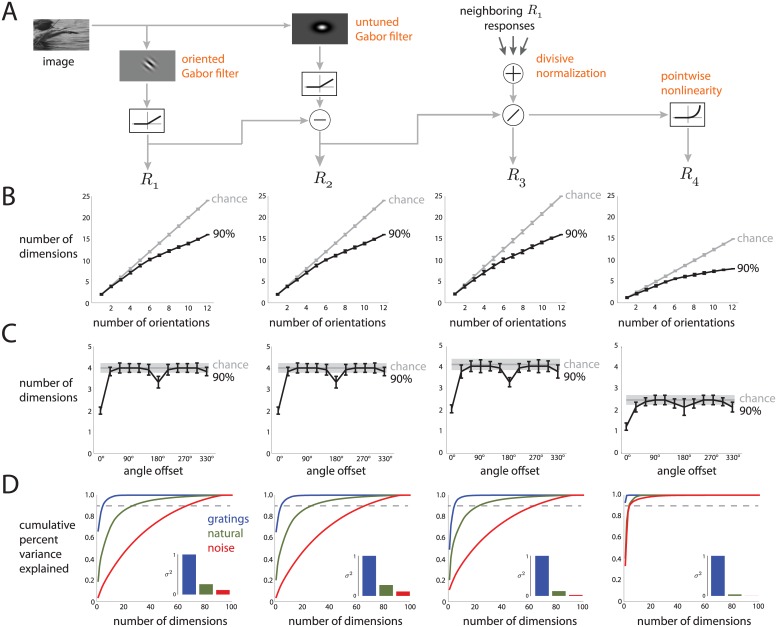
Dimensionality of model responses to individual gratings and movies. *A*: Block diagram of the RF model. We considered the activity in the model at four different components (*R*_1_, *R*_2_, *R*_3_, *R*_4_). *B*: Dimensionality of model activity versus number of orientations, computed in the same manner as in Fig 2*B*. *C*: Dimensionality of model activity versus angle offset between two orientations, computed in the same manner as in Fig 2*D*. *D*: Dimensionality and variance of model responses to movies, computed in the same manner as in Fig 3*C*. Results are based on 100 model neurons.

We first assessed the dimensionality of each component’s responses to the same individual gratings as presented to the monkeys. We observed similar trends between the population activity (Fig 2*B* and 2*D*) and the activity of the RF model (Fig 6*B* and 6*C*). As expected, the model captures key aspects of the population response to gratings, including direction selectivity. However, the last component of the model (“pointwise nonlinearity”) showed substantially smaller dimensionalities than the other components (Fig 6*B* and 6*C*, rightmost panels). This decrease in dimensionality is due to the exponential function exaggerating the anisotropy of the distribution of the model activity. For example, if the distribution of the responses across images resembles an ellipsoid in the 100-dimensional firing rate space, the exponential function would expand the variances of the major axes considerably more than the variances of the minor axes. The major axes would explain a greater proportion of the overall variance, resulting in fewer dimensions. Because we selected the values of the exponents independently from other model parameters, the discrepancy in dimensionality for the last component might not be present in the original model in [25], whose parameters were fit together. Still, our results indicate dimensionality can be sensitive to some nonlinear transformations.

Next, we assessed the dimensionality of each component’s responses to the same movie stimuli as presented to the monkeys. The ordering of dimensionality for each component of the model (Fig 6*D*) followed that of the population activity (Fig 3*C*). As for the individual gratings, the pointwise nonlinearity substantially reduced the dimensionality of the model activity. A discrepancy between the model and the population activity was the ordering of variance: each component of the model exhibited greater variance for the gratings movie than for the natural movie (Fig 6*D*, insets), but this was not the case for population activity (Fig 3*C*, insets). This discrepancy stems from the oriented Gabor filters in the first component of the model. Because the temporal frequency of these filters was matched to that of the drifting sinuosoid gratings, the filters were modulated strongly by the gratings movie.

### Dimensionality of model outputs to parametrically-varied stimuli

One advantage of working with an RF model is that we can assess the dimensionality of the responses to images that were not shown to the monkeys. With the caveat that the RF model might not capture aspects of the responses of real neurons, we assessed how the dimensionality of each component’s outputs varies as we parametrically alter the visual stimuli.

**Fig 7:**
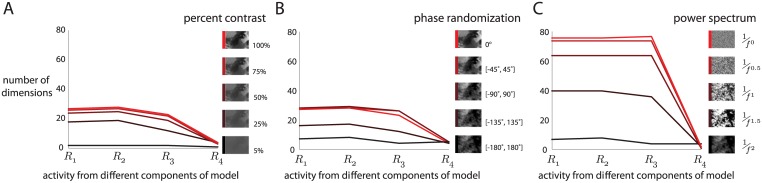
Dimensionality of model responses to parametrically-altered versions of the images in the natural movie. *A*: Images were generated by gradually decreasing contrast of the original images. *B*: Images were generated by adding a random offset to the phases of the original images, which transformed the natural images to pink noise. Each random offset was drawn from a uniform distribution over the specified range. *C*: Images were generated by raising the power spectrum of the same pink noise images generated in *B* to a fractional exponent. This gradually transformed the pink noise images to white noise.

We first considered varying the contrast of the natural movie images. Initially, one might think that contrast would have no effect on dimensionality because each dimension in pixel space is scaled equally. However, this is not the case for two reasons. First, before scaling, each image was re-centered by subtracting out the image’s mean pixel intensity—not the mean pixel intensity across all images. Thus, changing the contrast is not a simple scaling across all images. The second reason is that there are nonlinearities in the model (e.g., the linear rectifying functions), which can “warp” the scatter of data points (where each point corresponds to an image), thereby changing the dimensionality. When we varied the contrast of the natural movie images, we found that dimensionality decreased with decreasing contrast (different colors in Fig 7*A*). This decrease in dimensionality occurs because, as contrast decreases, the mean luminance dominates each image. At a contrast of 0%, the images lie along a line in pixel space (i.e., the [1, 1, …,1] direction).

Next, we randomized the phases of each natural image by adding a random offset to each phase value. The offsets were drawn from uniform distributions of varying extent. We considered small offsets drawn from the range [−45°, 45°] to large offsets drawn from the range [−180°, 180°]. When the phases were completely random (i.e., a range of [−180°, 180°]), the statistics of the images were akin to pink noise [26]. We observed that as the extent of the phase randomization increased, the dimensionality decreased (Fig 7*B*). Although randomizing the phases removed higher-order spatial correlations (e.g., edges and textures), it did not remove the spatial correlations brought about by the low frequencies of the power spectrum, which are strongly represented in natural images [27]. Only a small number of dimensions are needed to capture these low-frequency spatial correlations, because most of the pixels inside the region covered by the RFs of the model’s filters have pixel intensities that co-vary strongly.

Finally, to remove these low-frequency spatial correlations, we gradually transformed each phase-randomized image (i.e., pink noise image) to a white noise image by raising the power spectrum of the pink noise images to a fractional exponent. As the power spectrum of the pink noise images became more similar to white noise, the dimensionality increased (Fig 7*C*, black to red). This increase is not because the model neurons were more responsive to white noise images than pink noise images. Instead, the outputs of the model expressed more activity patterns for the white noise images due to the uncorrelated pixel intensities. This is consistent with the dimensionality trends of the population activity, where we observed that the population response to white noise had the highest dimensionality of the three movies but the lowest amount of variance (Fig 3*C*).

Based on these results, the model predicts that lowering contrast and randomizing the phases of a set of natural images will decrease the dimensionality of the population response. We also observed that for all manipulations of the visual stimuli, the outputs of the last component of the model (pointwise nonlinearity) showed substantially lower dimensionality than outputs at other model components, consistent with the results in Fig 6. This suggests that certain nonlinearities (e.g., pointwise exponentiation) affect dimensionality more than others (e.g., divisive normalization).

### Dimensionality in different layers of a deep neural network

**Fig 8:**
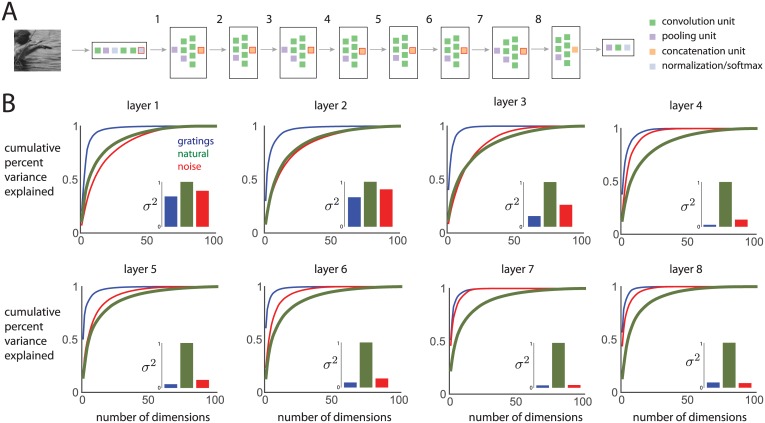
Dimensionality of activity in different layers of a deep convolutional neural network (CNN). *A*: The CNN comprised different layers (black outline boxes), where each layer comprised a group of units that performed specific operations, such as convolution, pooling, concatenation, and normalization. For each of the red-outlined units, we assessed the dimensionality of the activity of 100 filters. *B*: Dimensionality and variance of model responses in each layer to movies, computed in the same manner as in Fig 3*C*.

To build intuition about how the dimensionality of population activity might change at different stages of visual processing, we examined a deep convolutional neural network that was previously trained with over 1 million natural images and showed high accuracy on a well-known image recognition challenge [28]. The deep network takes an image as input, processes the image through layers of filtering and nonlinear operations, including convolution, pooling, and normalization (Fig 8*A*). The earlier layers capture low-level image statistics, such as oriented edges, while the deeper layers capture high-level image statistics, such as features that distinguish a car from a building [28]. This hierarchical processing resembles the processing found in the visual system, and indeed, the progressive layers of deep networks appear to mimic the progressive processing stages of the ventral stream [29]. We used this deep network to assess how dimensionality changes from one layer to the next, providing a prediction of how dimensionality might change in different visual areas along the ventral stream.

We input the same movie stimuli that we presented to the monkeys into the deep network, and examined the dimensionality of the filter outputs in different layers of the deep network (Fig 8*B*). For each layer, we analyzed the 100 filters that had RFs closest to the center of the image. We found that the earliest layer (Fig 8*B*, layer 1) showed dimensionality and response variance trends that matched the V1 population activity (Fig 3*C*). In progressively deeper layers (Fig 8*B*, going from layers 1 to 8), the responses to the gratings and noise movies decreased in dimensionality and relative variance, whereas the responses to the natural movie increased in dimensionality and relative variance. These findings are consistent with our understanding of the ventral stream: in progressive stages of visual processing, neurons become more sensitive to features in natural images and less sensitive to artificial images [30]. These results thus provide a prediction of how the dimensionality and variance of trial-averaged population responses to natural and artificial images should change along the ventral stream, which can be tested in future experiments [17].

## Discussion

To aid in understanding the outputs of dimensionality reduction, we chose to study a brain area close to the sensory periphery (V1). This allowed us to vary the sensory inputs and ask whether the outputs of dimensionality reduction change in a sensible way. By applying PCA to trial-averaged population responses to different classes of visual stimuli, including sinusoidal gratings, a natural movie, and white noise, we found that the dimensionality of the population responses grows with the stimulus complexity. In addition, we assessed whether the population responses to different stimuli occupy similar dimensions of the population firing rate space using a novel statistical method (the pattern aggregation method). We found that the population responses to stimuli as different as gratings and natural movies tended to occupy similar dimensions. For comparison, we applied the same analyses to the activity of a recently-proposed V1 receptive field model and a deep convolutional neural network, both of which showed trends similar to the real data. We further used these models to predict the dimensionality trends of the population responses to visual stimuli not shown to the monkeys, as well as the dimensionality trends of population responses in brain areas other than V1.

Many previous studies have compared visual cortical responses to natural and artificial stimuli on a single-neuron level (e.g., [20–22, 31, 32]). Their predominant approach was to define a parameterized RF model to relate a neuron’s activity to the visual stimulus. These studies found that, although RF models derived from natural stimuli share properties with those derived from artificial stimuli [20, 31], there can be important differences [20–22]. Here, rather than relating each neuron’s activity to the stimulus, we relate the activity of the recorded neurons to each other. We can then ask how this relationship (i.e., the covariation of trial-averaged activity among neurons) changes for different classes of visual stimuli. This approach has been adopted for pairs of neurons (i.e., signal correlation) [33], and we extend this work to characterize the signal correlation among all pairs of neurons at once. We found that the gratings, natural, and noise stimuli elicit many common basis patterns, consistent with previous studies showing similarities between RFs measured with gratings and natural stimuli [20] and those measured with natural and noise stimuli [31]. Finally, our finding of some unique basis patterns for each stimulus class suggests that estimates of RFs will best capture the responses to the same type of stimulus used in estimating the RFs, as reported in previous studies [21, 22].

The quality of most RF models has been evaluated based on their ability to predict the activity of individual neurons (e.g., [20–23, 25, 31]). Given that there can be, in some cases, a substantial difference between the predictions of RF models and the recorded neural activity [21, 22, 31], especially for natural scenes, we need to quantify how they are different in an effort to improve the RF models. This is often quantified by computing the percent variance of the recorded activity explained by the model for each neuron individually [21–23]. Our work provides a complementary way to compare RF models and recorded activity by examining the entire population together. We can compare the many V1 models that have been proposed by assessing which ones best reproduce the relative dimensionalities across stimuli and the similarity of basis patterns observed in recorded activity. The model that we tested does reproduce the dimensionality trend of the population activity across stimuli, but does not reproduce the response variance trend (Fig 6*D*). We speculate that a different spatiotemporal filter in the first component of the model can help to increase the response variance to natural images [21], thereby better matching the response variance trend of the model to that of the recorded activity.

Different basis patterns may be used by the population activity during different task epochs, suggesting that certain basis patterns drive downstream areas more effectively than others [16, 34]. Thus, the identification of which basis patterns are used may be critical for understanding how different brain areas interact on a population level [35]. Furthermore, the activity patterns across a neural population have been used to study normalization [36], decision making [2, 37], learning [4], and motor planning [5]. In the present work, we have developed a statistical framework (the pattern aggregation method) to measure the similarity of basis patterns across any number of stimulus conditions or time points. We validated this framework using recordings in V1, and the framework can be applied broadly to other brain areas.

The measurement of dimensionality depends on many factors, including the choice of dimensionality reduction method, the number of neurons (cf. S1 Fig), and the number of data points. In principle, one should use a nonlinear dimensionality reduction method (e.g., [38–40]) because the underlying manifold of the population activity is likely to be nonlinear. For example, divisive normalization nonlinearly maps the tuning curves of a population of neurons onto a high-dimensional sphere [41], and a nonlinear dimensionality reduction method may be able to extract the lower-dimensional embedding. However, most studies using dimensionality reduction in systems neuroscience have focused on linear methods [1–4, 6, 11, 12, 16, 42]. The reasons are that 1) most nonlinear methods rely on a dense sampling of the population activity space, in contrast to experimental data which tend to sparsely sample the space, and 2) it is usually difficult to assess the contribution of each neuron to a low-dimensional space identified by nonlinear methods. For the latter reason, we would not be able to compare how similar are the patterns for different stimuli, as we do in this study. Despite these caveats, we applied a nonlinear method, fractal dimensionality [17, 38], to the three movies and their population responses (S3 Fig). The ordering of fractal dimensionality across stimuli was consistent with that of PCA dimensionality (Fig 3). Together with the results showing how the dimensionality ordering of the population activity depends on stimulus complexity (Figs 2*B* and 3*C*) and neuron count (S1 Fig), this finding indicates that a linear method can provide useful insights, even if the underlying manifold is indeed nonlinear.

There are several other factors that can affect the dimensionality of population responses. Dimensionality can depend on the properties of the particular neurons being sampled. In V1, these properties include the size and scatter of the receptive fields, as well as their preferred phases, orientations, and spatial frequencies. Another factor that can affect dimensionality in V1 is the size of the visual stimulus. We presented large visual stimuli that extended outside of the classical RF of most neurons. Previous studies have shown that stimulation outside of the classical RF tends to increase the sparseness of V1 responses [43–46], which may affect the dimensionality of the population response. Sparseness leads to independence in the responses between neurons [43, 44, 47], and may lead to increased discriminability of the population activity [48]. Independence implies that each basis pattern only captures modulations of a single neuron (i.e., only one element of each pattern is non-zero), and the dimensionality (as assessed by PCA) depends on the relative variances captured by the basis patterns (in this case, the relative variances of the neurons). To our knowledge, there is no general relationship between sparseness and dimensionality. For all these reasons, it is not possible to make absolute statements about the dimensionality of V1. Instead, we made relative comparisons where all of the factors affecting dimensionality are fixed, except for the stimulus content.

Although we believe that the results shown here are representative of a wide range of gratings, natural, or noise stimuli, they should be interpreted in the context of the particular visual stimuli used. For example, the dimensionality of the gratings movie and its population response could be increased by including more than one spatial and temporal frequency. Similarly, the dimensionality of the natural movie and its population response likely depends on the particular movie clip shown. If the scenes in the movie change more quickly (or slowly) over time, then we would expect the dimensionality over a 30-second time window to be larger (or smaller). For the noise movie, our results in Fig 5*B* indicate that showing more instances of white noise is not likely to further increase the dimensionality of the population response. However, changing the statistics of the noise in the pixels could change the dimensionality of the population response (Fig 7*C*).

At a population level, several studies have compared visual cortical activity evoked by natural and artificial stimuli to spontaneous activity [49–51]. These studies focused on the raw neural activity, which includes both the trial-averaged component (i.e., the PSTHs) and trial-to-trial variability. Here, we focused on the trial-averaged component. Because trial-to-trial variability can be substantial relative to the trial-averaged component [52, 53], it is difficult to directly compare results of these previous studies to those reported here. The current study can be extended to study the population structure of trial-to-trial variability using a dimensionality reduction method such as factor analysis rather than PCA [13] in tandem with the pattern aggregation method.

For the stimuli that we tested and the recordings we made, we found that the dimensionality trends were consistent between the visual stimuli and the population responses. However, this need not be the case for other visual stimuli and other brain areas. In fact, a dimensionality trend that is inconsistent between the stimuli and responses may yield important insight into how the stimuli are encoded by the neurons under study. Part of resolving this potential discrepancy may relate to the way in which stimulus complexity is measured. PCA dimensionality captures only a particular aspect of the stimulus, namely the anisotropy of the distribution of pixel intensities in an Euclidian space. Other aspects of the stimuli may influence the neural responses more strongly, and alternate measures of stimulus complexity can be used, for example fractal dimensionality (S3 Fig) [17, 38] or a method based on image features extracted by a deep neural network (Fig 8). Future studies employing additional stimuli and brain areas can elucidate whether dimensionality trends remain consistent between sensory stimuli and population responses.

Our work lays a solid foundation to assess the dimensionality and similarity of basis patterns of neural population activity. Because V1 is a well-studied brain area and is close to the sensory input, our results can be compared with expectations based on our intuition and well-established RF models. Moving forward, these methods can be applied broadly to other brain areas and behavioral tasks to examine how the complexity of the population response changes due to conditions such as attentional state, learning, and contextual modulation.

## Materials and Methods

### Neural recordings

Details of the neural recordings have been described previously [54, 55]. Briefly, we recorded from primary visual cortex (V1) of anesthetized, paralyzed macaque male monkeys. Anesthesia was administered throughout the experiment with a continuous intravenous infusion of sufentanil citrate (6–18 *μ*g/kg/hr). Eye movements were minimized with a continuous intravenous infusion of vecuronium bromide (100–150 *μ*g/kg/hr). Experiments typically lasted 5–7 days. All experimental procedures followed guidelines approved by the Institutional Animal Care and Use Committee of the Albert Einstein College of Medicine at Yeshiva University, and were in full compliance with the guidelines set forth in the US Public Health Service Guide for the Care and Use of Laboratory Animals.

Neural activity was recorded using 96-channel multi-electrode arrays (Blackrock Microsystems, Salt Lake City, Utah), which covered 12.96 mm^2^ and had an electrode length of 1 mm. The electrodes were inserted to a nominal depth of 0.6 mm to confine recordings mostly to layers 2–3. Recordings were performed in parafoveal V1, with RFs within 5 degrees of the fovea. Voltage waveform segments that passed a separately-chosen threshold for each channel were later spike-sorted offline. For comparisons of population responses to different orientation gratings, we spike sorted responses together across all orientations, thereby obtaining a common set of units. Similarly, we spike sorted responses together across all three movies. We included sorted units for which the voltage waveform had a signal-to-noise ratio (SNR) greater than 1.5, where SNR is defined as the ratio of the average waveform amplitude to the standard deviation of the waveform noise [56]. This SNR threshold yielded both single-unit and multi-unit activity (see [57] for comparison of these signals) with a median SNR near 2.5 for all datasets. We analyzed only neurons with mean firing rates greater than 1 spike per second.

### Visual stimuli

We used two sets of visual stimuli. The first set (termed the individual gratings set) consisted of individual presentations of drifting sinusoidal gratings with different orientations. The second set (termed the movies set) consisted of different classes of visual stimuli, including a sequence of drifting sinusoidal gratings with different orientations, a contiguous sequence of natural scenes, and white noise. All stimuli were presented on a CRT monitor with a frame rate of 100 or 120 Hz, and had mean luminance of approximately 40 cd/m^2^. We used a look-up table for all stimuli to correct for the nonlinearity between input voltage and output luminance in the monitor.

#### Individual gratings

Full-contrast (100%) drifting sinusoidal gratings with 12 equally-spaced orientations (30° between adjacent orientations, covering 360°) were presented. We use “orientation” to refer to the angle of drift and “direction” to refer to two orientations that are 180° apart (i.e., opposing drift) [19]. Spatial frequency (1.3 cpd) and temporal frequency (6.25 Hz) were chosen to evoke robust responses from the population as a whole, and the position and size (8–10 degrees) were sufficient to cover the RFs of all recorded neurons. Gratings were block-randomized across the 12 orientations and presented for 1.28 seconds each, followed by a 1.5 second inter-trial interval consisting of an isoluminant gray screen. We conducted 200 trials for each of the 12 orientations.

#### Movies

We presented three different 30-second grayscale movies: gratings, natural, and noise, as described previously [55]. Each movie comprised 750 unique images (each presented for four consecutive video refreshes of the CRT, for an effective framerate of 25 Hz). The movie frames were surrounded by a gray field of average luminance. The mean (normalized) RMS contrasts were 0.17, 0.17, and 0.09 for the gratings, natural, and noise movies, respectively. Thus, the global statistics were matched for the gratings and natural movies.

The gratings movie was a pseudo-randomly chosen sequence of full-contrast drifting sinusoidal gratings with 98 equally-spaced orientations (3.67° between adjacent orientations, covering 360°). The presentation of each drifting grating of a particular orientation lasted 300 ms. Spatial frequency (1.3 cpd), temporal frequency (6.25 Hz), position and size (8 degrees or 640 pixel diameter circular aperature) were chosen to evoke high firing rates and to sufficiently cover RFs of all recorded neurons. Two 300 ms periods of blank screen frames were included at a randomly-chosen position in the sequence, bringing the total to 100 stimuli in a block lasting 30 seconds. A 30-second movie with the same random sequence of gratings and blank screens was repeated 120 times.

The natural movie was a 30 second consumer film of a contiguous sequence of natural scenes converted to grayscale (a monkey wading through water). The movie was displayed in a square of 5 degrees (400 × 400 pixels) to cover all RFs, and repeated 120 times.

The noise movie was 30 seconds of white noise, where each 4 degree (320 × 320 pixel) frame comprised a 40 × 40 grid of 8-pixel squares. Each 8 × 8-pixel square displayed a random intensity drawn from a uniform distribution independently (in space and time) of other squares. The entire 40 × 40 grid was randomly shifted or “jittered” *k* pixels (1 ≤ *k* ≤ 8) horizontally and vertically between consecutive frames to avoid high-contrast grid effects. One noise movie was randomly generated, and the same movie was repeated 120 times.

### Preprocessing of population activity and visual stimuli

#### Population activity for individual gratings

We presented the individual gratings stimulus set to three monkeys (101r, 102l, 103r). After removing neurons that did not satisfy the SNR and firing rate criteria, the lowest number of neurons across monkeys was 61. Because measurements of dimensionality can be influenced by the size of the recorded population, we selected a random subset of 61 neurons from the data sets with larger populations, in order to combine results across monkeys (dimensionality trends were similar across monkeys for all analyses). We considered neural activity in a 1 second window starting at stimulus onset (discarding the remaining 0.28 seconds of response). Results were similar for a 1 second window starting 100 ms after stimulus onset to avoid onset transient effects. For each neuron and orientation, we took spike counts in 20 ms bins and averaged them across trials to create a peri-stimulus time histogram (PSTH), yielding 50 time points. Because a processing step in PCA subtracts from each PSTH the mean across the 50 time points, we can compare (across different orientations) fluctuations of trial-averaged activity around its mean.

#### Population activity for movies

We presented the movies stimulus set to two monkeys (monkey 1: 103l, 61 neurons, monkey 2: 102l, 81 neurons). For each neuron, we took spike counts in 20 ms bins during the 30-second movie presentation and averaged them across trials to create a PSTH. For each movie, this yielded a PSTH with 1,500 time points for each neuron. PCA then centers each PSTH by subtracting its mean, enabling the study of fluctuations of the firing rate around the mean. We ensured the neural activity was centered just once for all analyses (i.e., for non-overlapping one-second time windows, the “local” mean was not subtracted again). In this way, all dimensionality measurements are made using a common reference frame, thereby allowing comparison of dimensionality across different time windows (analysis in Fig 5*B*).

#### Visual stimuli for movies

To relate neural complexity to stimulus complexity, we performed PCA on the movie stimuli. This required the following preprocessing steps in order to match the sizes of images across stimuli and remove incidental spatial correlations in the noise movie. The processed movies were only used for PCA analysis; the original, unprocessed movies were shown to subjects. We first cropped the grating and natural image frames to 320 x 320 pixels to match the size of the noise images. The noise movie had two incidental spatial correlations from the experimental design that prevented it from being “true” white noise: spatial correlations caused by noise images being comprised squares of 8 × 8 pixels with identical intensities and spatial correlations caused by jittering the squares to avoid grid effects. To weaken these correlations, we converted the images of all movies to 8 × 8 pixel squares. The average pixel intensity was computed for each pixel square, and each image was compressed from a matrix of 320 × 320 values to a matrix of 40 × 40 values. Because we averaged over fixed pixel squares, we did not eliminate all incidental spatial correlations in the noise movie, as the borders between the 8 × 8 pixels with identical intensities were jittered randomly from frame-to-frame. Thus, the red eigenspectrum curve in Fig 3*B* is not perfectly diagonal, which is expected from true white noise. Each 40 × 40 pixel image was then reshaped into a vector (with size 1,600 × 1). Each movie stimulus was therefore represented as 750 vectors (one for each image) in a 1,600-dimensional space, where each axis corresponds to the average intensity of one pixel block.

### Assessing dimensionality and similarity of patterns

#### Assessing dimensionality

We describe here how we assessed the dimensionality of the population activity and that of the visual stimuli. Conceptually, the dimensionality is the number of basis patterns needed to describe the population activity (Fig 1) or the pixel intensities of the visual stimuli. There are many ways to assess dimensionality, including linear [58] and nonlinear [39, 40] methods. Here, we focus on the most basic linear dimensionality reduction method, principal component analysis (PCA). PCA is appropriate for use with trial-averaged neural activity because trial averaging removes much of the Poisson-like spiking variability, consistent with PCA’s implicit assumption of no observation noise [13]. PCA has also been applied to pixel intensities in previous studies [17, 27].

PCA dimensionality was assessed as the number of basis patterns needed to explain 90% of the variance in the population activity or visual stimuli. The threshold of 90% is arbitrary, and we verified that the results were qualitatively similar for other high-percentage thresholds (e.g., 70% and 80%). It is possible to use a data-driven approach to determine the variance threshold [17]. Under this approach, data which require a small or large number of dimensions to explain the majority of the variance can be deemed low-dimensional. To aid in interpreting dimensionality comparisons, we chose to use a pre-determined (90%) variance threshold in this work. It is important to note that the dimensionality depends on many factors, including the dimensionality reduction method (here, PCA), metric for assessing dimensionality (here, 90% variance explained), the number of neurons, and the number of data points used. Thus, we do not attempt to interpret the dimensionality in an absolute sense. Rather, we make relative comparisons of dimensionality across experimental conditions, while keeping these other factors fixed.

#### Assessing similarity of patterns

In addition to computing the number of basis patterns needed for each condition, we sought to compare the patterns across conditions. The following is the intuition for performing this comparison. Consider two sets of patterns: condition *A* requires *k*_*A*_ patterns and condition *B* requires *k*_*B*_ patterns, where *k*_*A*_ > *k*_*B*_. At one extreme, the space spanned by the condition *A* patterns includes the space spanned by the condition *B* patterns (i.e., the spaces overlap). In this case, the joint space is described by *k*_*AB*_ = max(*k*_*A*_, *k*_*B*_) = *k*_*A*_ patterns. At the other extreme, the space spanned by the condition *A* patterns is orthogonal to the space spanned by the condition *B* patterns. In this case, the joint space is described by *k*_*AB*_ = *k*_*A*_ + *k*_*B*_ patterns. In general, *k*_*AB*_ will lie between max(*k*_*A*_, *k*_*B*_) and *k*_*A*_ + *k*_*B*_. The closer *k*_*AB*_ is to max(*k*_*A*_, *k*_*B*_), the more similar the patterns are. Conversely, the closer *k*_*AB*_ is to *k*_*A*_ + *k*_*B*_, the more dissimilar the patterns are.

To compute *k*_*AB*_, we tried several different approaches. In the first approach, we aggregated the population activity (or visual stimuli) for different conditions, then applied the same PCA method to find the number of dimensions that explained 90% of the variance. A problem with this approach is that it does not account for possibly different variances of the population activity (or visual stimuli) across conditions. The consequence is that *k*_*AB*_ obtained by this method can be less than max(*k*_*A*_, *k*_*B*_), thereby violating the intuition laid out above. To see this, consider a scenario where *k*_*A*_ > *k*_*B*_ and condition *B* has much larger variance than condition *A*. The aggregated population activity (or visual stimuli) would be dominated by condition *B*, essentially ignoring the patterns of condition *A*. As a result, the aggregated dimensionality *k*_*AB*_ would be close to *k*_*B*_, which is less than max(*k*_*A*_, *k*_*B*_) = *k*_*A*_. This motivated us to consider a second approach in which we normalized the population activity (or visual stimuli) such that the direction of greatest variance was 1 for each condition. However, there are still scenarios for which *k*_*AB*_ obtained by this method is less than max(*k*_*A*_, *k*_*B*_), due to how the variance is distributed across patterns.

#### Pattern aggregation method

To overcome the issues described above, we developed an alternative approach which guaranteed that *k*_*AB*_ would be between max(*k*_*A*_, *k*_*B*_) and *k*_*A*_ + *k*_*B*_. This method, termed the pattern aggregation method, is based on first identifying the *k*_*A*_ patterns for condition *A* (represented by *U*_*A*_: *N* × *k*_*A*_, whose columns are orthonormal and where *N* is the number of pixels or the number of neurons) and *k*_*B*_ patterns for condition *B* (represented by *U*_*B*_: *N* × *k*_*B*_, whose columns are orthonormal) separately using PCA. Then, we aggregate the patterns into a single matrix *V* = [*U*_*A*_*U*_*B*_] (where *V*: *N* × (*k*_*A*_ + *k*_*B*_)) and designate *k*_*AB*_ to be the effective column rank (defined below) of *V*. In the case where *k*_*A*_ > *k*_*B*_ and the space spanned by the condition *A* patterns includes the space spanned by the condition *B* patterns, then *k*_*AB*_ will be max(*k*_*A*_, *k*_*B*_) = *k*_*A*_. On the other hand, if the space spanned by the condition *A* patterns is orthogonal to the space spanned by the condition *B* patterns, then *k*_*AB*_ will be *k*_*A*_ + *k*_*B*_. For more than two conditions, this method is easily extended by aggregating the patterns for all conditions into *V* = [*U*_*A*_*U*_*B*_*U*_*C*_…].

**Fig 9:**
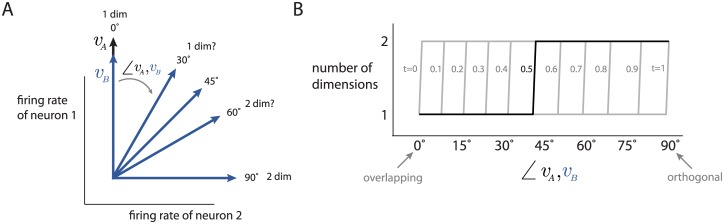
Assessing similarity of basis patterns. *A*: Conceptual illustration for two neurons, where *v*_*A*_ denotes the basis pattern for one condition and *v*_*B*_ denotes the basis pattern for another condition. As *v*_*B*_ is rotated away from *v*_*A*_, the question is at which point do we consider *v*_*A*_ and *v*_*B*_ to span two dimensions. *B*: The transition from one to two dimensions in *A* depends on the rank threshold *t*. For *t* = 0.5, the transition occurs when the angle between *v*_*A*_ and *v*_*B*_ is near 45 degrees.

The effective column rank of *V* is the number of “large” singular values of *V*. The definition of “large” requires setting a threshold (termed the rank threshold, between 0 and 1) for the singular values. The rank threshold determines how different two basis patterns (cf. Fig 1, green) need to be before they define separate dimensions. To gain intuition for the rank threshold, we ran a simulation where we rotated a 2-d unit vector (*v*_*B*_) from a position of overlap with another unit vector (*v*_*A*_, 0°) to a position of orthogonality (90°) (Fig 9*A*). We varied the rank threshold for different angles between *v*_*A*_ and *v*_*B*_, and assessed the effective column rank of *V*. A rank threshold *t* = 0 means that slight deviations of *v*_*B*_ away from *v*_*A*_ lead to *V* = [*v*_*A*_*v*_*B*_] having an effective column rank of two (Fig 9*B*). In other words, if two patterns are slightly different, the dimensionality of the data will be two. On the other hand, a rank threshold *t* = 1 means that *v*_*B*_ needs to be orthogonal to *v*_*A*_ for *V* to have an effective column rank of two (Fig 9*B*). In other words, two patterns need to be orthogonal for the dimensionality of the data to be two; else, the dimensionality will be one. As a compromise between the two extremes, we used a rank threshold *t* = 0.5 throughout this study. This threshold means that the transition from one to two dimensions occurs when the angle between *v*_*A*_ and *v*_*B*_ is near 45 degrees (Fig 9*B*). We also verified that this intuition holds for higher-dimensional spaces by rotating a subspace towards an orthogonal subspace by taking a convex combination of the two subspaces and measuring the rank at intermediate rotations.

#### Computing dimensionality expected by chance

To assess whether two sets of patterns are similar or dissimilar, it is necessary to compare *k*_*AB*_ to a chance level, rather than simply asking whether *k*_*AB*_ is closer to the minimum dimensionality bound max(*k*_*A*_, *k*_*B*_) or maximum dimensionality bound *k*_*A*_ + *k*_*B*_. The reason is that the chance level depends on the relative values of *k*_*A*_, *k*_*B*_, and *N*, and does not simply lie halfway in between max(*k*_*A*_, *k*_*B*_) and *k*_*A*_ + *k*_*B*_. For example, if *N* is large relative to *k*_*A*_ and *k*_*B*_, the chance level would lie near *k*_*A*_ + *k*_*B*_ because randomly drawn patterns in a high-dimensional space tend to be orthogonal. We compute the chance level by drawing patterns randomly from the *N*-dimensional space, and compute a distribution of aggregated dimensionalities. Specifically, we repeatedly draw *k*_*A*_ orthonormal patterns and *k*_*B*_ orthonormal patterns in an *N*-dimensional space, and measure their aggregated dimensionality using the same method as applied to the data.

The above method makes the implicit assumption that all patterns within the *N*-dimensional space can be shown in the data. In some cases, there is reason to believe that not all patterns in the *N*-dimensional space can be achieved. For example, the underlying neural circuitry may constrain the space of activity patterns that a population of neurons is capable of producing [4, 10]. In such settings, the chance level should be computed by drawing patterns randomly from an *M*-dimensional space, where *M* < *N*. Ideally, the range of *M* should be determined using a large number of stimuli and/or time points to obtain as accurate an estimate of the space of activity patterns that can be produced as possible. We can then assess whether the results hold for all values of *M* within this range.

#### Similarity index

The similarity of two sets of patterns depends, relative to the two extremes (max(*k*_*A*_, *k*_*B*_) and *k*_*A*_ + *k*_*B*_), on whether *k*_*AB*_ is larger or smaller than the chance level. We summarized this dependence with a single number, the similarity index. The similarity index is defined as $s=\frac{{\hat{k}}_{AB}-k_{AB}}{k_{A}+k_{B}-\text{max}(k_{A},k_{B})}$. Intuitively, we take the difference between the mean chance dimensionality (${\hat{k}}_{AB}$) and the actual dimensionality for the aggregated patterns of *A* and *B* (*k*_*AB*_). Then, we normalize this difference by the difference between the two extreme dimensionalities, yielding an index between −1 and 1. A similarity index of *s* > 0 means that the patterns are more similar (i.e., more overlapping) than expected by chance, whereas *s* < 0 means that the patterns are less similar (i.e., closer to orthogonal) than expected by chance.

### Statistical assessment of dimensionality

Error bars for the dimensionality of the population activity were computed by subsampling from all time points. We chose subsampling over bootstrapping, since bootstrapping led to biased estimates due to small sample size relative to the number of neurons. For population responses to the stimulus movies, we randomly subsampled 750 of the 1,500 time points, computed the dimensionality of the subsampled points, and repeated this 100 times (Fig 4*C*). Similarly, for the dimensionality analysis in 1 second windows, we randomly sampled 25 of the 50 time points 100 times (Fig 5*A*). We did not compute error bars for the visual stimuli because subsampling was not possible (only 750 available time points), and bootstrapping was not possible due to the small number of time points relative to the dimensionality (1,600) of the pixel space. All *p*-values were computed from 10^5^ runs of a random permutation test.

### Receptive field model

We considered a recently-proposed RF model of V1 neurons [25] that includes four components—oriented Gabor filtering, subtraction of untuned suppressive filtering, divisive normalization, and pointwise nonlinearity—common to many RF models of V1 neurons [23]. We input the individual gratings and movie stimuli into the model, and asked how the dimensionality ordering after each model component compares to that of the population activity. The parameter values for 100 model neurons were drawn from the distributions reported in [25] rather than fit to data because we did not present the mixed gratings stimuli necessary for fitting the parameters. For this reason, we compare dimensionality trends between the model and population activity, rather than their absolute values.

#### Component 1: Oriented Gabor filtering

The first component for each model neuron is an oriented Gabor filter applied to the input image. Because [25] incorporates direction selectivity only when parameterizing the orientation tuning curve of a neuron, we needed a way to incorporate temporal filtering that would be applicable to any image sequence and allow for direction selectivity. We adopted a straightforward approach to extend the 2-d Gabor filter to a 3-d Gabor filter, which considers both space and time [59]. The following equation describes the 3-d Gabor filter *G* for pixel location (*x*, *y*) and time index *t*:

G(x,y,t)=real{H(x,y,t)·S(x,y,t)} (1)

where

$$\begin{array}{ccc} H\left(x,y,t\right) & = & \exp\left[-\frac{1}{2}\left({\tilde{x}}^{2}+{\tilde{y}}^{2}+{\tilde{t}}^{2}\right)\right]\\ S\left(x,y,t\right) & = & \exp\left[-j2\pi (\gamma_{s}x^{\prime }+\phi +\gamma_{t}t^{\prime })\right] \end{array}$$

and $\left[\tilde{x},\tilde{y},\tilde{t}\right]$ and [*x*′, *y*′, *t*′] are defined below. Thus, the Gabor filter is the product of a Gaussian envelope *H* and a sinusoid *S*. To orient the Gabor, the pixel locations (*x*, *y*) are first rotated by orientation angle *θ*. To incorporate direction selectivity, (*x*, *y*, *t*) are further rotated by angle *β*, which is either 0° or 180°. These rotations are achieved with a rotation matrix, *R*:

$$R=\left[\begin{array}{ccc} 1 & 0 & 0\\ 0 & \cos(\beta ) & -\sin(\beta )\\ 0 & \sin(\beta ) & \cos(\beta ) \end{array}\right]\times \left[\begin{array}{ccc} \cos(\theta ) & -\sin(\theta ) & 0\\ \sin(\theta ) & \cos(\theta ) & 0\\ 0 & 0 & 1 \end{array}\right]$$ (2)

One can then compute the rotated [*x*′, *y*′, *t*′]^*T*^ = *R* × [(*x* − *x*_loc_), (*y* − *y*_loc_), *t*]^*T*^ and the scaled and rotated $[\tilde{x},\tilde{y},\tilde{t}{]}^{T}=R\times [(x-x_{\text{loc}})/\sigma_{x},(y-y_{\text{loc}})/\sigma_{y},t/\sigma_{t}{]}^{T}$, where (*x*, *y*, *t*) are scaled by (*σ*_*x*_, *σ*_*y*_, *σ*_*t*_) before rotation to align the Gaussian envelope with the orientation angle. The Gabor filter *G* is normalized by its Frobenius norm.

We chose the parameter values for each model neuron in the following manner to be biologically plausible. The center of the RFs (*x*_loc_, *y*_loc_) were drawn from a Gaussian with mean 160 and variance 20 to be partially overlapping at the center of the 320 × 320 pixel image. The spatial phase *ϕ* was drawn from a uniform distribution between 0 and 2*π*. The temporal frequency *γ*_*t*_ = 6.25 Hz was fixed at the same temporal frequency as that for the drifting gratings. Orientation angle *θ* was drawn from a uniform distribution between 0 and *π*. The direction of drift *β* was randomly chosen from the set {0, *π*}. The sizes of the Gaussian envelope were *σ*_*x*_ = 20 + |*ϵ*| (where *ϵ* was drawn from a standard Gaussian) and *σ*_*y*_ = *cσ*_*x*_ (where *c* is the aspect ratio drawn from the distribution reported in [25]). The size of the temporal envelope was *σ*_*t*_ = 8*d*, where *d* ∈ [0, 1] is the direction selectivity constant found in [25]. The spatial frequency *γ*_s_ was drawn from the distribution reported in [25].

To compute the output of this model component at timestep *t* and considering the past *T* = 15 timesteps, the dot product of the 3-d Gabor filter (*G*) with the sequence of images (*I*_*t*, *t* − 1, …, *t* − *T*_) was computed as: $L_{1}=\sum_{m=t-T}^{t}\sum_{x,y}G(x,y,m-t)I_{m}(x,y)$. This quantity was then passed through a linear rectifying function to yield the response *R*_1_ = max(0, *L*_1_).

#### Component 2: Subtraction of untuned Gabor filtering

The next component of the model subtracts from *R*_1_ the response of an untuned suppressive filter to the current image. The untuned suppressive filter *G*_untuned_ was computed as a difference of 2-d Gaussians:

$$G_{untuned}\left(x,y\right)=G_{\sigma_{1}}\left(x,y\right)-G_{\sigma_{2}}\left(x,y\right)$$ (3)

$$\text{where }G_{\sigma }=\frac{1}{2\pi \sigma^{2}}\text{exp}\left[-\frac{1}{2}\left(\frac{{\left(x-x_{\text{loc}}\right)}^{2}}{\sigma^{2}}+\frac{{\left(y-y_{\text{loc}}\right)}^{2}}{\sigma^{2}}\right)\right]$$ (4)

The RF centers (*x*_loc_, *y*_loc_) were kept the same as those for the first model component, and the spreads of the filters were fixed at *σ*_1_ = 20, *σ*_2_ = 30. The dot product of the untuned Gabor filter *G*_untuned_ and the current image *I*, computed as $L_{2}=\sum_{x,y}G_{\text{untuned}}(x,y)I(x,y)$, was then passed through a weighted linear rectifying function *S*_2_ = max{0, *ωL*_2_}, where *ω* was drawn from the distribution reported in [25]. Finally, the output of this model component *R*_2_ was the difference of the response of the untuned Gabor filter with the previous component’s output: *R*_2_ = *R*_1_ − *S*_2_.

#### Component 3: Divisive normalization

The next component of the model is divisive normalization, which divides the output of the previous component with the mean output of all other model neurons from the first component plus an additive offset. Let $R_{1}^{i}$ and $R_{2}^{i}$ refer to the output of the *i*th model neuron from the first and second model components, respectively. Then, the output of the *i*th model neuron after divisive normalization $R_{3}^{i}$ was computed as follows:

$$R_{3}^{i}=\frac{R_{2}^{i}}{\sigma_{\text{offset}}+\frac{1}{N-1}\sum_{\begin{array}{c} j=1,\\ j\neq i \end{array}}^{N}R_{1}^{j}}$$ (5)

where the additive offset *σ*_offset_ was drawn from the distribution reported in [25], and the number of neurons *N* = 100.

#### Component 4: Pointwise nonlinearity

The final output of the model was computed by passing the output of the previous model component through a pointwise nonlinearity:

$$R_{4}={\left[\max\{0,R_{3}\}\right]}^{q}$$ (6)

where the exponent *q* was drawn from the distribution reported in [25].

### Parametrically altering the visual stimuli

To study how dimensionality of the model outputs changes as we parametrically alter the visual stimuli, we performed three analyses. First, we varied the contrast of the images of the natural movie in 5 different increments: 100%, 75%, 50%, 25%, 5%. For each image, we subtracted the mean luminance of that image, scaled the result by one of the percentages above, and added back the mean luminance. Second, we transformed the images of the natural movie to pink noise by randomizing the phase of the 2-d Fourier transform for each image. To avoid removing local contrast of the natural images [26], we added an offset to each phase, where the offset was randomly drawn from a uniform distribution over the range [−*α*, *α*]. We considered 5 values for *α*: 0° (i.e., no change of the natural image), 45°, 90°, 135°, 180° (i.e., pink noise). Third, we transformed the pink noise images (which retained the power spectrum of the natural images) to white noise by raising the power spectrum of the pink noise to different fractional exponents. The intuition behind this is that the power spectrum of natural images falls off as 1/*f*^2^, where *f* is frequency [27]. Raising the power spectrum to a fractional exponent, for example 1/2, transforms the 1/*f*^2^ fall-off to a 1/*f* fall-off. To ensure the average magnitude of the power spectrum was similar for each transformed image, we normalized the power spectrum of each image by dividing by its sum of magnitudes *λ*. We then raised the normalized power spectrum to 5 different exponents: 1 (i.e., pink noise), 3/4, 1/2, 1/4, 0 (i.e., white noise). Finally, we scaled the resulting power spectrum by *λ*.

### Deep convolutional neural network

To assess how the ordering of dimensionality might change at different stages of visual processing, we studied a deep convolutional neural network (CNN). We used an instantiation of a CNN called GoogLeNet [28], trained with the Princeton Vision and Robotics Toolkit [60], and available in Matlab with MatConvNet [61]. The CNN had different processing units, including convolution, pooling, concatenation, and normalization/softmax. Each unit comprised many filters (from 10^3^ to 10^6^) that performed the same operation (e.g., convolution) but on different spatial regions of the input. Each layer comprised a group of units (shown in Fig 8*A*). We assessed dimensionality of the outputs of eight consecutive layers of the deep network. For the first layer, we analyzed the outputs of 100 filters of the second normalization unit. For layers 2 to 8, we analyzed the outputs of 100 filters of the concatenation units. For each analyzed unit, we chose the 100 filters to have the closest RFs to the center of the image.

## Supporting Information

S1 Fig The ordering of dimensionality of the population responses to the movie stimuli remained consistent for a wide range of neuron counts. We randomly subsampled a smaller number of neurons from the 61 neurons of monkey 1 (left panel) and from the 81 neurons of monkey 2 (right panel). We then computed the dimensionality for the subsampled population responses to each movie separately. We assessed dimensionality as the number of dimensions needed to explain 90% of the variance. Error bars represent the standard deviation for 50 different subsamples.
(EPS)

S2 Fig Two-dimensional projections for the visual stimuli and population activity. Projections were found using the DataHigh software [62], to show variance within each movie and separation between movies. *A*: Projection for the pixel intensities for the visual stimuli, capturing 18% of the total variance. Each dot is an image, and *v*_1_ and *v*_2_ are orthonormal projection vectors of the high-dimensional pixel space. *B*: Projections for the population responses for monkey 1 (left panel, 14% of the total variance) and monkey 2 (right panel, 27% of the total variance). Each trajectory traces out the population activity timecourse for one movie (gratings: blue, natural: green, noise: red). The orthonormal projection vectors *v*_1_ and *v*_2_ of the high-dimensional firing rate spaces are computed separately for each monkey.
(EPS)

S3 Fig Fractal dimensionality of the visual stimuli and population responses. Fractal dimensionality was computed by the same method as in [17]. The intuition behind fractal dimensionality is to define a hypersphere with radius *r* and ask how the number of points *N* contained within that sphere grows as *r* increases. The faster *N* grows with *r*, the higher the dimensionality. We plot log(*N*) versus log(*r*), where the slope is defined to be the fractal dimensionality. *A*: Fractal dimensionality of the pixel intensities of the gratings (blue), natural (green), and noise (red) movies. Slopes (Δ) were computed by linear regression between the bounds of 6 and 12 of the log number of neighbors. *B*: Fractal dimensionality of the population responses to the gratings, natural, and noise movies for monkey 1 (left panel) and monkey 2 (right panel). Slopes were computed by linear regression between the bounds of 4 and 10 of the log number of neighbors.
(EPS)
